# On Peritonitis, Simulating Perforation of the Intestines

**Published:** 1854-02

**Authors:** 


*On Peritonitis, simulating perforation of the intestines.*
By Dr. Thirial.
(Translated for the Examiner.)

The opinion inculcated by Louis, that perforation of the intestine has taken place whenever, in an acute disease, a violent local pain in the abdomen suddenly takes place, which soon spreads, is increased by pressure, and is accompanied by anxious and distressed expression of the features, and straining and vomiting, is liable, according to the author, to many exceptions, of which the following are instances.

In October, 1835, a girl, aged 21 years, was admitted into La Charité, with symptoms of mild typhoid fever. After about 20 days of illness, she became convalescent and commenced taking a little food, when suddenly, in consequence of great mental agitation, she was seized with the most violent pain in the abdomen, accompanied with bilious vomiting. Her features expressed great anxiety and her pulse sank. Rayer diagnosticated peritonitis, dependent upon perforation of the bowel, and applied on the following day 20 leeches. Large doses of opium, (25 ctgrmm. Extr. thebaic p. die), rest and prohibition of drink were also ordered. Notwithstanding these, the vomiting and the other symptoms continued; the tongue became dry; the abdominal pain having disappeared, excepting under strong pressure. In the evening, 72 hours after the onset of the attack, the patient died.

*Autopsy.* The peritoneum to some degree, was moderately injected, and upon its visceral surfaces, plastic exudations were presented in many places. In the upper pelvis, 4 or 5 oz. of a milky, albuminous fluid were found; the mesentery was covered throughout with a more or less thick pseudo-membrane of thin consistence. Water was poured through the whole intestinal canal without showing the smallest perforation. The whole length afterwards opened, exhibited the bowel perfectly sound, except that at the end of the ileum, and more particularly near the ileo-cœcal valve, 4 or 5 of Peyer's glands were found, which were of a blackish color, but showed no ulceration. The other organs of the abdomen were sound. Spleen small and firm. In the posterior part of the lung there was slight congestion.

2d. A laborer, 18 years old, tall and slender, entered the hospital on the 10th of February, 1846, with symptoms of severe typhoid fever; headache, diarrhœa, debility, &c., &c. Costiveness, swelling of the abdomen, nightly delirium, dull expression of countenance, scattered spots upon the face and abdomen, soon ensued and once a moderate epistaxis. From the 15th to the 20th of February all the symptoms increased, especially the delirium, which became violent; the tongue and teeth became black and coated with sordes; speech difficult; pains in the back, belly, and lower extremities; involuntary movement of the lips; subsultus tendinum, &c., &c. This state continued until the 25th, when a slight improvement took place: but at the same time deafness commenced in the right ear. The previous treatment consisted in bleeding, warm applications, clysters, cooling drinks, &c., &c. From this time tonics were given and two blisters were applied to the inner surface of the thighs. By the end of February the patient had recovered his appetite and was considered convalescent. About the 5th of March, quite a large discharge of healthy pus took place from his right ear, in consequence of an abscess there of the size of a nut. Two days afterwards another abscess on the right side of the abdomen, and soon afterwards several more on the back of the neck, thigh, &c. appeared; some of which broke and others were opened. During the night of the 30th of March, after an attack of diarrhœa, that had lasted several days, the patient was seized with a violent pain in the stomach, and vomiting of green matter. On the 31st his countenance was pale, features expressing pain and anguish; he rested upon his back, with his legs drawn up to his belly; the latter very painful under pressure. It appeared that during the preceding day, he had suffered from indigestion. He was ordered depletory and narcotic applications externally, and opiates by the mouth. On the 1st of April his thirst became insatiable; pain in the abdomen more violent; pulse frequent and small; respiration short and gasping. The pain increased more and more, and on the following day at noon he died.

*Autopsy.* Neither by the above mentioned experiment of filling the intestine with water, nor by the closest investigation of the opened intestine, could any perforation be discovered. At the end of the ileum, some of Peyer's glands slightly projected above the surface, but were otherwise normal. Others in the neighborhood of the valve projected more distinctly and were black in color. Two or three of them showed spots slightly ulcerated and cicatrised. The mucous membrane of the valve was colored slate-grey, and about one foot from the valve the mucous membrane of the small intestine was injected. Its walls were decidedly thinned. The large intestine showed nothing abnormal. The mesenteric glands were somewhat swollen. The coating of the peritoneum of the whole intestinal canal was reddened. The subserous vessels were injected. In the individual sulci of the convolutions was found a sero-purulent liquid. In the cavity of the lesser pelvis, a considerable deposit of yellow, thick, creamy pus, and in various parts of the peritoneum large amounts of adherent pseudo-membrane appeared. All the other organs were normal.

These cases, besides the interest which attaches to them as respects their diagnosis, are important as regards the question of the cure of peritonitis, dependent on perforation of the bowel. Stokes, Graves, and others report many instances of cures in these cases, which they attribute to large doses of opium. The author ventures to suggest from his observations, that most of these cases where there could be no post-mortem as the last and strongest proof, should be attributed, not to a perforation, but to a more or less circumscribed peritonitis. He allows, however, that the differential diagnosis of these affections is still imperfect.—(*Wagner,*) *Schmidt's Jahrbuch, from L' Union,* 83—85, 1853.

